# *BioTriplex*: a full-text annotated corpus for fine-tuning language models in gene-disease relation extraction tasks

**DOI:** 10.1093/bioinformatics/btag037

**Published:** 2026-01-21

**Authors:** Charlotte Collins, Panagiotis Fytas, İlknur Karadeniz, Huiyuan Zheng, Simon Baker, Ulla Stenius, Anna Korhonen

**Affiliations:** Language Technology Laboratory, Theoretical and Applied Linguistics, Faculty of Modern and Medieval Languages and Linguistics, University of Cambridge, Cambridge CB3 9DA, United Kingdom; Centre for Human Inspired Artificial Intelligence, Institute for Technology and Humanity, University of Cambridge, Cambridge CB2 1SB, United Kingdom; Language Technology Laboratory, Theoretical and Applied Linguistics, Faculty of Modern and Medieval Languages and Linguistics, University of Cambridge, Cambridge CB3 9DA, United Kingdom; Language Technology Laboratory, Theoretical and Applied Linguistics, Faculty of Modern and Medieval Languages and Linguistics, University of Cambridge, Cambridge CB3 9DA, United Kingdom; Department of Artificial Intelligence and Data Engineering, Özyeğin University, Istanbul 34794, Türkiye; Institute of Environmental Medicine, Karolinska Institutet, Stockholm 171 77, Sweden; Language Technology Laboratory, Theoretical and Applied Linguistics, Faculty of Modern and Medieval Languages and Linguistics, University of Cambridge, Cambridge CB3 9DA, United Kingdom; Institute of Environmental Medicine, Karolinska Institutet, Stockholm 171 77, Sweden; Language Technology Laboratory, Theoretical and Applied Linguistics, Faculty of Modern and Medieval Languages and Linguistics, University of Cambridge, Cambridge CB3 9DA, United Kingdom; Centre for Human Inspired Artificial Intelligence, Institute for Technology and Humanity, University of Cambridge, Cambridge CB2 1SB, United Kingdom

## Abstract

**Motivation:**

Automatic information extraction from biomedical texts requires machine learning methodology that can recognize biomedical entities, characterize inter-entity relationships, and relate extracted information to specific research topics. Large language models (LLMs) excel in general tasks but perform less reliably in the biomedical domain, where texts are characterized by extensive technical terminology and semantic variations from general literature. There is an unmet need for annotated full-text datasets that can be used to fine-tune language models for significant biomedical applications. Here, we focus on extraction of the complex relationships between genes and diseases.

**Results:**

We present *BioTriplex*, a corpus of 100 full-length biomedical research articles (comprising 604 subsection texts) manually annotated with disease names, genes, and 21 subtypes of disease–gene relationships. We employ *BioTriplex* to train the LLaMA 3.1 8B language model in gene–disease relation extraction. Our fine-tuned model outperforms zero-shot and few-shot approaches, both within the LLaMA 3.1 architecture and across the larger state-of-the-art LLMs GPT-4 and Claude Sonnet 3.7, and classifies gene–disease relation types with broader scope and greater granularity than previously described. These results validate *BioTriplex* as a useful full-text data resource and underscore the value of specialized datasets in fine-tuning language models for important biomedical tasks.

**Availability and implementation:**

https://github.com/PanagiotisFytas/BioTriplex

## 1 Introduction

Genetics plays a key role in disease causation, with some diseases resulting from a single gene mutation and others developing from interactions between multiple gene variants and environmental factors (Passarge 2021, Motsinger-Reif *et al.* 2024). In the field of biomedicine, the complex relationship between disease phenotypes and the ∼20 000 human protein-coding genes is researched intensively, generating an expansive literature (Przybyla and Gilbert 2022) that far exceeds the ability of any researcher to read manually (Landhuis 2016). To build on existing knowledge, researchers need automatic methods for extracting and interpreting the relationships reported between genes and diseases in biomedical texts. Natural language processing (NLP) has the potential to perform these tasks through using machine learning (ML) methodologies to turn unstructured text into structured data. NLP in the biomedical domain presents a particular challenge because of the diversity of disciplines involved and the specialized technical terminology used, which can reduce the generalizability of trained models (Galea *et al.* 2018, Luo *et al.* 2023). Annotated corpora can be used to train ML algorithms and generate domain-specific language representation models to perform named entity recognition (NER) to recognize a wide range of biomedical entities, and relation extraction (RE) to identify and extract semantic relationships between entities. Gene–disease RE has been explored using various specialized biomedical language models including BioBERT (Lee *et al.* 2020), BioREx (Lai *et al.* 2023), LORE (Li *et al.* 2024), RENET2 (Su *et al.* 2021), and BioRED (Luo *et al.* 2022, Lai *et al.* 2025). Large language models (LLMs) excel in the general domain, having excellent ability to handle large volumes of data and perform complex tasks, but often perform relatively poorly in specialized biomedical RE applications. LLM performance can be improved through fine-tuning on custom labelled datasets (Lai *et al.* 2023, Islamaj *et al.* 2024).

Development of improved methodology for biomedical NLP is limited by the availability of labelled datasets for training and fine-tuning models. Datasets can be labelled automatically, e.g. the TBGA gene–disease dataset (Marchesin and Silvello 2022), but automatic methods can be less accurate than labelling by human annotators (Zhao and Goto 2025) and may miss nuances of context. Manually-annotated datasets such as CRAFT, a corpus of full-text papers labelled with biomedical concepts (Bada *et al.* 2012, Verspoor *et al.* 2012), are valuable for training NLP models but are scarce, likely due to the significant time and domain-specific expertise required to generate them (Kühnel and Fluck 2022, Luo *et al.* 2023). Existing manually-annotated corpora of gene–disease relations, e.g. BioRED (Luo *et al.* 2022) and RENET2 (Su *et al.* 2021), are restricted to article abstracts and consequently exclude important information present within other paper subsections. They also make only limited distinctions between the many different subtypes of relations, restricting potential to link extracted information to specific research questions.

Here, we address the unmet need for fine-grained, manually annotated corpora based on complete articles. *BioTriplex* is a new corpus of 100 full-text biomedical research papers (comprising 604 individual paper subsections) manually annotated with three classes of biomedical entities: *Genes*, *Human Diseases* and 21 subtypes of gene-disease *Relations*. *Biotriplex* (i) includes abbreviations and acronyms, (ii) is annotated with 21 different subtypes of gene–disease relationships, relating to different research questions, and (iii) distinguishes between background and novel information. We evaluate the *BioTriplex* corpus by using it to train an open-source LLM, LLaMA 3.1 (Grattafiori *et al.* 2024), in the gene–disease RE task, achieving substantially improved task performance over the non-fine-tuned model and over two larger LLMs.

## 2 Materials and methods

### 2.1 Corpus annotation

#### 2.1.1 Literature retrieval

We downloaded CC BY-licensed full-text articles from the PubMed Central open-access database. Articles were filtered to select 10 000 papers in which the abstract contained at least one mention of a gene (name, symbol, or synonym), as listed on the GeneCards human gene database (https://www.genecards.org) and at least one mention of a human disease, as listed in the European Molecular Biology Organization (EMBO) Human Disease Ontology (https://www.ebi.ac.uk/ols4/ontologies/doid). One hundred articles were randomly selected for annotation and processed into separate text files for each relevant section. Figures, Authors, Affiliations, References, and Acknowledgement sections were not used.

#### 2.1.2 Terminology

**Figure 1:**
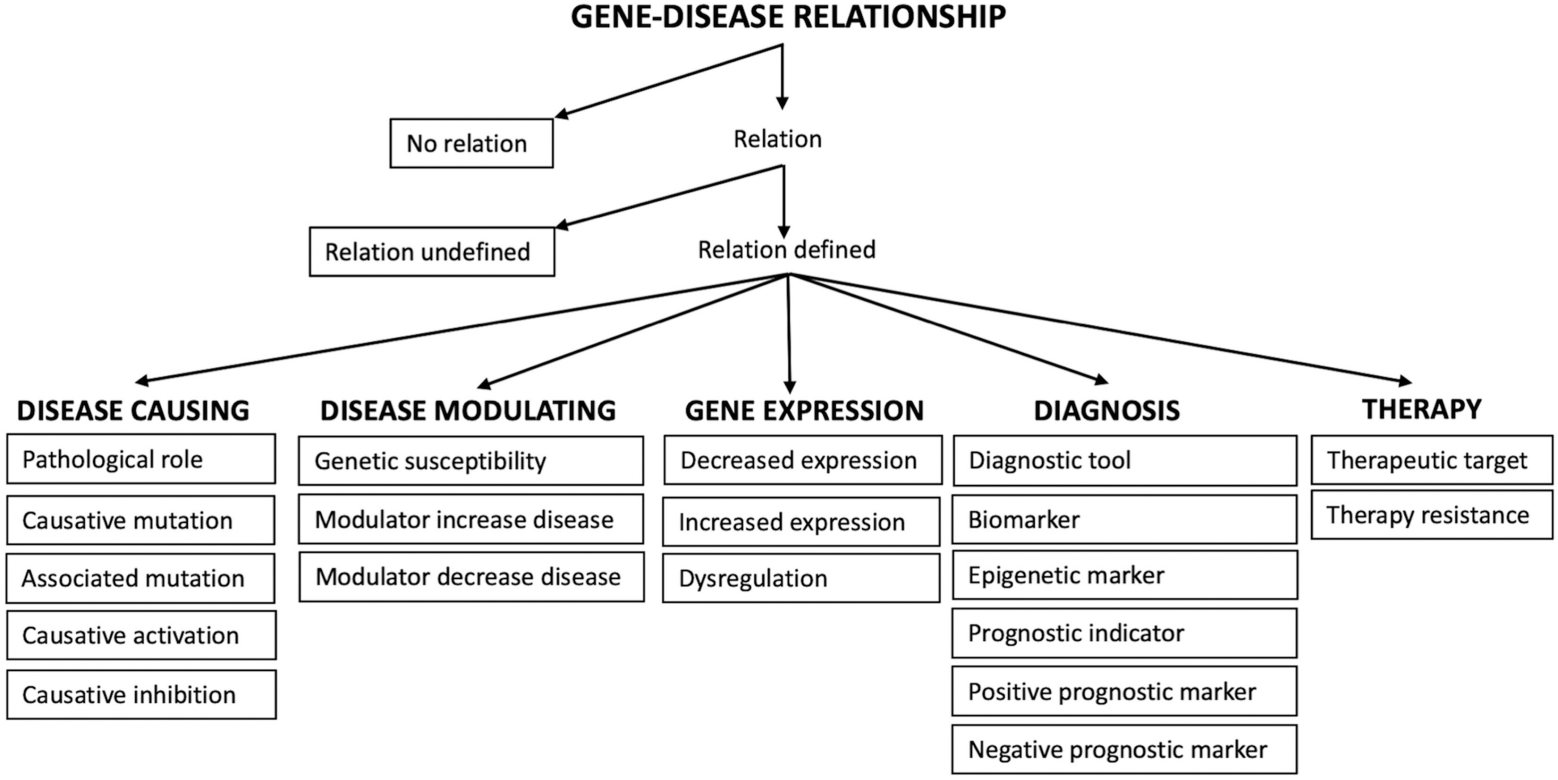
Ontology of gene–human disease relation types occurring in texts. Terms within boxes represent the categories of annotated relation type.

**Figure 2:**
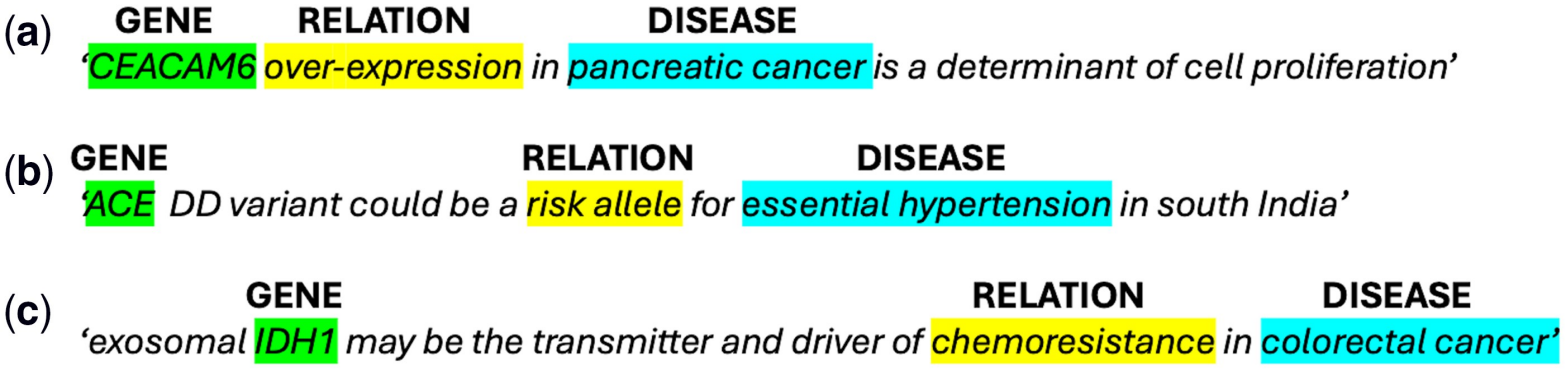
Marking and linking related entities in texts. Each highlighted entity forms part of a gene–disease–relation linked triplet. ‘Genes’ are highlighted in green, ‘human diseases’ are highlighted in blue, and ‘relations’ are highlighted in yellow. Relations correspond to ontology terms as follows: (a) ‘increased expression’; (b) ‘genetic susceptibility’; (c) ‘therapy resistance’.

To systematically extract the key elements of descriptions of disease to gene relationships from biomedical texts, we focused on three categories of entity: ‘genes’, ‘human diseases’, and ‘relations’. We marked all occurrences of human diseases and genes. Relations were only marked where they described the relationship between a marked human disease and a marked gene. ‘Genes’ were defined as exact matches to a gene name, symbol, or synonym as listed in the Human Gene Database (https://www.genecards.org/). ‘Human diseases’ were defined as exact matches to any disease listed in the EMBO Human Disease Ontology (https://www.ebi.ac.uk/ols4/ontologies/doid). We also marked abbreviated forms of matched diseases. In the EMBO Human Disease Ontology, diseases are grouped into eight different categories: ‘disease by infectious agent’, ‘disease of anatomical entity’, ‘disease of cellular proliferation’, ‘disease of mental health’, ‘disease of metabolism’, ‘genetic disease’, ‘physical disorder’, and ‘syndrome’. Some diseases fall into two or more categories. ‘Relations’ were defined as phrases that (i) described the relationship between a named gene and a named human disease (defined above) and which (ii) were semantically related to one of the terms included in our Ontology of Relation Types (Figs. 1 and 2).

#### 2.1.3 Creation of an ontology of gene–human disease relation types

In preliminary studies, we carried out a series of test annotations of relevant papers to generate a list of common types of gene–disease relation present in biomedical texts. Our list was also inspired by a previous study in which seven types of disease–gene relationship were described (Milosevic and Thielemann 2022). We defined a list of 21 categories of gene–human disease relations: ‘no relation’, ‘relation undefined’, ‘pathological role’, ‘causative activation’, ‘causative inhibition’, ‘causative mutation’, ‘modulator decrease disease’, ‘modulator increase disease’, ‘biomarker’, ‘associated mutation’, ‘dysregulation’, ‘increased expression’, ‘decreased expression’, ‘epigenetic marker’, ‘therapy resistance’, ‘prognostic indicator’, ‘negative prognostic marker’, ‘positive prognostic marker’, ‘therapeutic target’, ‘diagnostic tool’, and ‘genetic susceptibility’. ‘No relation’ was used to label text that explicitly reported the absence of a relationship between a marked Gene and a marked Disease. ‘Relation undefined’ was used to label text in which a gene–disease relationship was reported but its nature was not specified. The 19 specific types of gene–disease relation were categorized into five main groups and used to build the ontology shown in Fig. 1.

#### 2.1.4 Annotation of texts

Annotation was carried out by a biologist with 9 years of postgraduate experience in cell biology experimental research. The multi-annotation environment (MAE) annotation tool (version 2.2.11) was used for the task (Stubbs 2011, Rim 2016) (http://keighrim.github.io/mae-annotation/). A custom DTD file was created to define the task framework. Annotation was carried out at the level of paper subsections (Abstract, Introduction, Methods, Results, Discussion, Conclusion, and other sections where present). Words or phrases were marked when they corresponded to one of the terms of interest described in Section 2.1.2. The MAE tool allows the addition of labels, or ‘assertions’, to marked entities. In all three categories, each marked entity was labelled as either ‘background’, defined as text describing previous work or existing knowledge, or as ‘result’, defined as text describing study methods, findings or conclusions. Entities in the human diseases category were additionally labelled with the EMBO human disease ontology categories to which they belonged, as described in Section 2.1.2, and entities in the relations category were additionally labelled according to the type of gene–disease relationship to which they belonged, as described in Section 2.1.3 and in our ontology (Fig. 1). We created link tags to record the links within triplets of marked entities (one gene, one human disease, and one relation) that formed the description of a relationship between a gene and a disease. Where a description involved more than one gene, disease, or relation type, we created a different triplet link tag for each relationship. Examples of marking and labelling text are shown in Fig. 2. Data 1, available as supplementary data at *Bioinformatics* online, provides further details of the annotation scheme.

To evaluate the precision of the annotation process, a secondary annotator with postgraduate cell biology experience undertook independent annotation of the Discussion sections from 25 articles, representing 1491 marked entities (6.6% of total in corpus) of which 100 were relation type entities (13.0% of total in corpus). Our definition of agreement required that annotators select exactly the same text spans and also assign the same entity type. We calculated values for the *F*1 metric (following its standard formulation), where a maximum value of 1 indicates perfect inter-annotator agreement. The overall *F*1 value for text marking across all three categories was 0.89, indicating high agreement and therefore high reliability of the annotation process. Individually, the *F*1 value for entities in the ‘genes’ category was 0.91 (‘high agreement’), for entities in the ‘human diseases’ category was also 0.91 ‘(high agreement’), and for entities in the ‘relations’ category was 0.71 (‘moderate agreement’).

### 2.2 ML methodology

#### 2.2.1 Dataset preprocessing

Each text was divided into segments with maximum lengths of 4000 characters. The dataset was split into training, validation, and test sets, using a 70:15:15 ratio. Stratified sampling was performed to ensure a balanced distribution of entities across dataset splits (Cochran 1977). To prevent data leakage, stratification was applied at the paper level, such that all text segments from the same paper were assigned to the same split. All relation types, including rarer ones, were represented in the test set, enabling comprehensive intrinsic evaluation. The distribution and total counts of the relation and entity types in the *BioTriplex* splits are shown in Data 3, available as supplementary data at *Bioinformatics* online. Detailed statistics on the variability of relation-type spans across splits are provided in Data 6, available as supplementary data at *Bioinformatics* online.

#### 2.2.2 RE and NER model baselines

**Figure 3:**
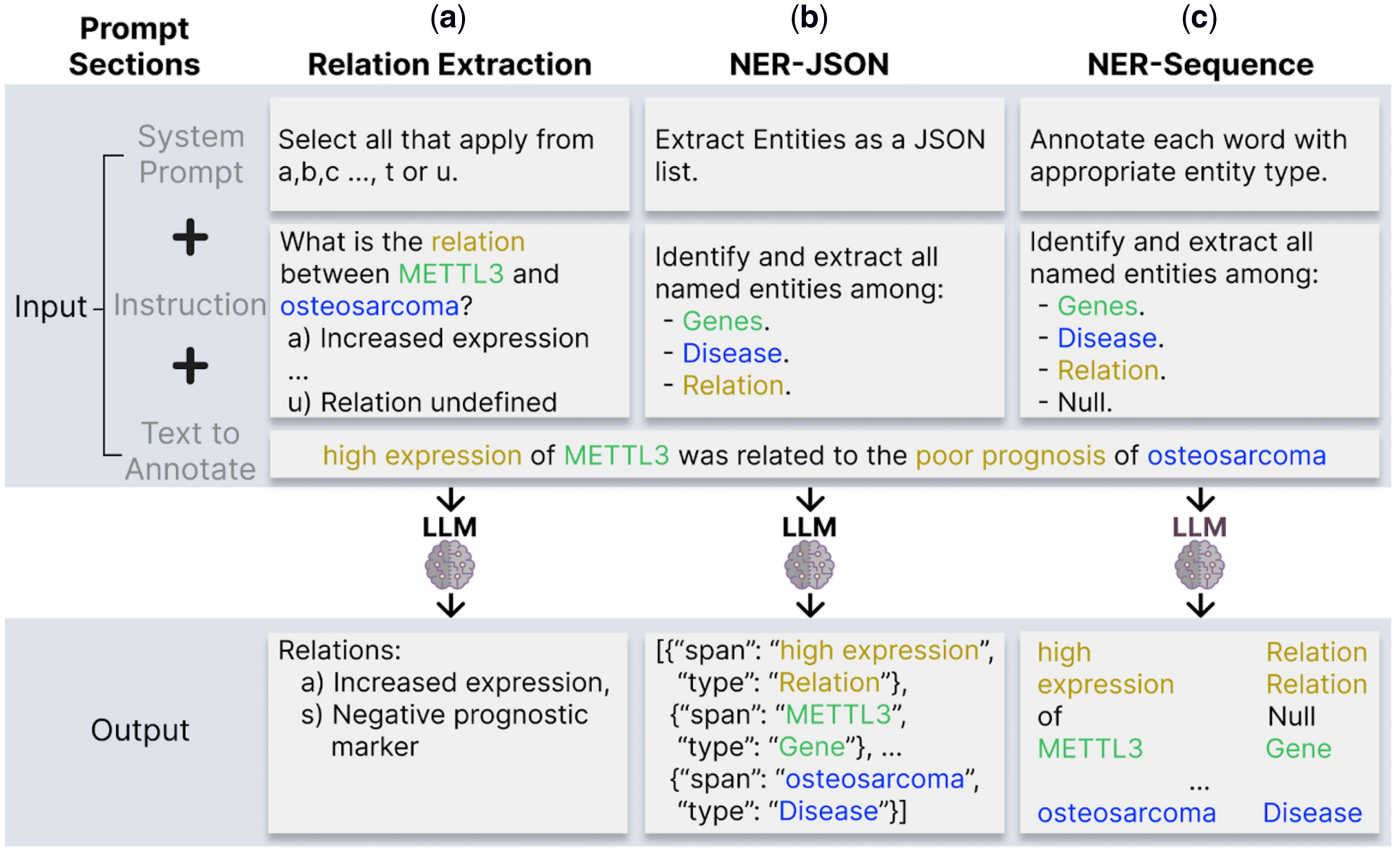
Overview of the RE and NER tasks in *BioTriplex* for LLM baselines. The input is a biomedical text and instructions that specify one of three subtasks: (a) extraction of the gene–disease relation based on a predefined set of relation types, (b) extraction of named entities (gene, disease, and relation) in JSON format, and (c) token-level annotation of each word with its corresponding entity label. Full details of prompts are provided in the codebase.

For the gene–disease RE task, we explored the performance of three modern LLMs: GPT-4 (OpenAI *et al.* 2023), Claude 3.7 Sonnet (Anthropic 2024; https://claude.ai) and LLaMA 3.1 8B (Grattafiori *et al.* 2024). We adopted a generative formulation for RE by prompting models with a multiple-choice question to identify the correct relation types between a gene and a disease. This formulation is extended from prior bidirectional transformer approaches, such as BioBERT (Lee *et al.* 2020) and BioREx (Lai *et al.* 2023), and aligns naturally with the generative capabilities of autoregressive language models. For comparison, we additionally evaluated the encoder-based models BioBERT and PubMedBERT (Gu *et al.* 2022), selecting the optimal decision thresholds for converting sigmoid outputs into discrete relation predictions using the validation set. We explored the zero-shot and few-shot performance of GPT-4, Claude 3.7 Sonnet, and LLaMA 3.1 8B, and performed parameter-efficient fine-tuning through low-rank adaptation (LoRA) (Hu *et al.* 2021) of LLaMA 3.1 8B. In the zero-shot setting, models were prompted to perform the task without seeing any task-specific examples, relying solely on a natural language instruction describing the task. In the few-shot setting, we adopted a 5-shot configuration, where each prompt includes five representative examples sampled from the annotation guidelines (Data 1, available as supplementary data at *Bioinformatics* online) to illustrate the task and the expected output format (Fig. 3).

For NER of genes, diseases, and relations, we generated two fine-tuned bidirectional transformer baselines: (i) PubMedBERT (Gu *et al.* 2020) within the DyGIE++ framework (Wadden *et al.* 2019), and (ii) BioBERT (Lee *et al.* 2020). In addition, we evaluated PubTator 3.0 (Wei *et al.* 2024) as a pre-annotated baseline for gene and disease NER. For LLaMA 3.1 fine-tuning, we experimented with two prompting approaches: instructing the LLM to generate a JSON-formatted list of entity spans and types (NER-JSON) or prompting it to output the sequence of words in the text paired with its corresponding entity type tag (NER-Sequence) (Fig. 3). All tables contain results for the same fixed seed. Details of the hyperparameters, fixed seeds, and model weights can be found at https://github.com/PanagiotisFytas/BioTriplex.

## 3 Results

### 3.1 Analysis of annotated dataset

#### 3.1.1 Corpus size and statistics

Our corpus of 100 full-text research papers comprised in total 604 subsections, 21 745 sentences and 586 781 words, with an average sentence length of 27.0 words (Table 1). Every research paper included an Abstract, and most also included Introduction, Methods, Results, and Discussion sections. Only 35 papers included a Conclusion subsection. Subsections present in only a small number of papers, e.g. ‘Case presentation’ and ‘Additional information’, were grouped together under the category ‘Others’ (comprising >6% of total words). The Results subsections contained the greatest volume of text and the Conclusion subsections contained the smallest volume (Table 1).

**Table 1:** Total numbers of texts, words, and sentences in the corpus by paper subsection.^a^

	Total texts	Total words	Total sentences	Words per text	Sentences per text	Words per sentence
Abstract	100	29 380	1177	294	12	25.0
Introduction	92	66 679	2245	725	24	29.7
Methods	89	138 031	5237	1551	59	26.4
Results	87	189 232	7177	2175	82	26.4
Discussion	94	124 055	4492	1320	48	27.6
Conclusion	35	5174	177	148	5	29.2
Others	107	34 230	1240	320	12	27.6
Total	604	586 781	21 745	971	36	27.0

a ‘Others’ represents pooled data from subsection types that were present in only a small number of papers.

#### 3.1.2 Frequency of occurrence of entities and linked triplets in different paper subsections

The total number of marked entities in the corpus was 22 970; this comprised 15 056 genes, 7133 human diseases, and 781 relations. The total number of linked triplets (one gene, one human disease, and one relation) was 1217, of which 3% crossed sentence boundaries.

**Figure 4:**
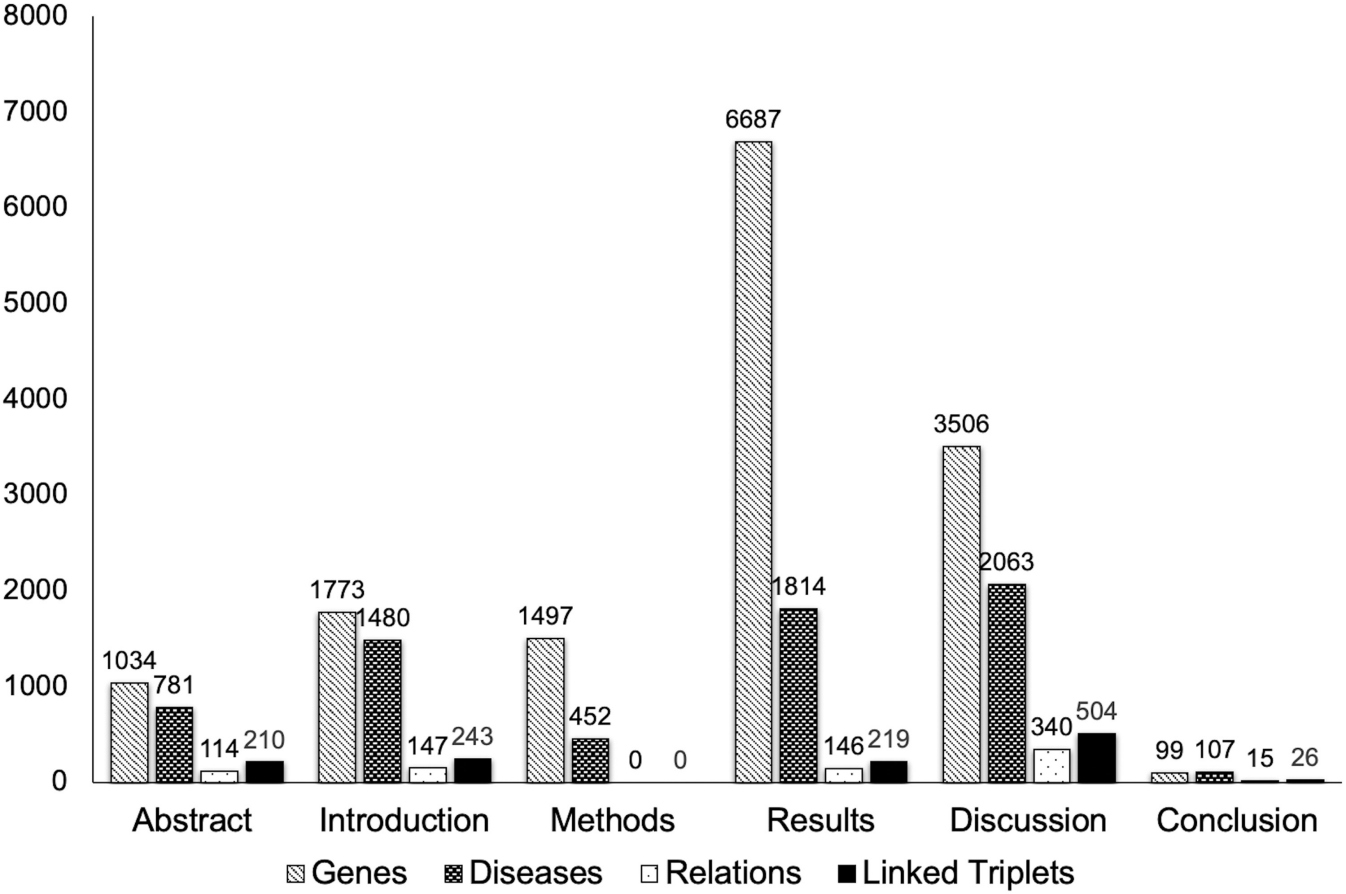
Total numbers of marked entities and linked triplets in different paper subsections.

The distribution of marked entities varied between different paper subsections, reflecting differing mean subsection lengths and also different types of content (Table 1; Fig. 4). The largest total number of entities was marked in the Results sections, correlating with their highest total word count. The largest numbers of marked human diseases and relations, and correspondingly, gene–disease–relation linked triplets, occurred in the Discussion sections. The Abstracts, Introductions, and Results sections contained broadly similar numbers of relations, which in each case were less than half the number recorded in the Discussion sections; the numbers of linked triplets followed the same pattern. The Methods sections did not contain any marked relations or linked triplets. The Conclusion sections contained low numbers of entities and triplets, reflecting the smaller number of texts with shorter average length (Table 1; Fig. 4).

#### 3.1.3 Distribution of marked entities between background information and novel findings

**Figure 5:**
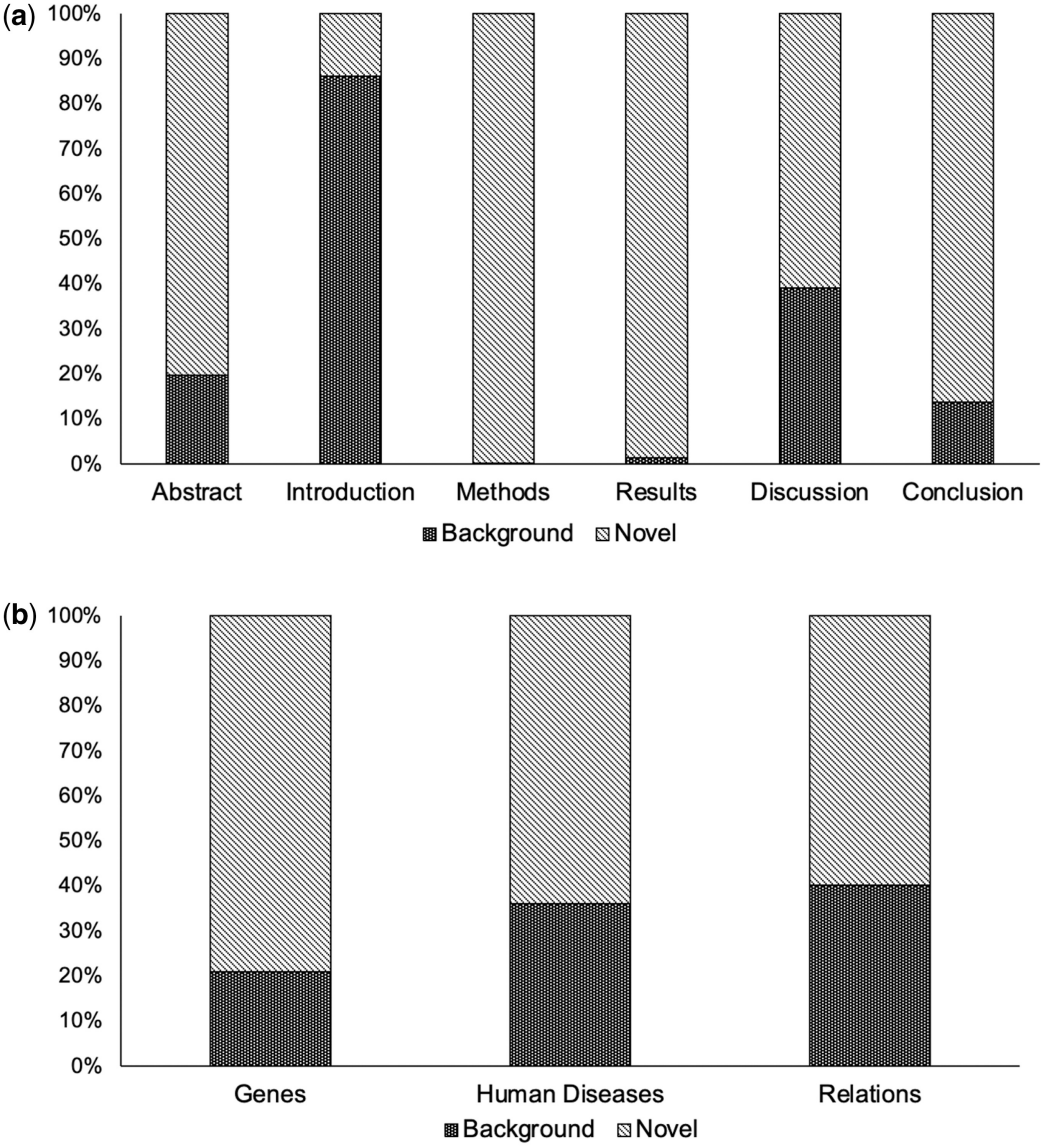
Percentages of background entities (previous work or existing knowledge) and novel entities (methods employed, findings, or conclusions) in the corpus, by (a) paper subsection and (b) entity category.

It is important that NLP methodology has the ability to distinguish between background and novel text because the same background information may be recycled through multiple research studies, leading to a risk of bias. In *BioTriplex*, each marked entity was therefore labelled to distinguish it as either background or novel, of which both were represented within all three entity categories. The highest percentages of novel entities were marked in the Methods and Results sections and the lowest percentage was marked in the Introduction sections (Fig. 5).

#### 3.1.4 Analysis of human disease–gene–relation linked triplets

All relation types described in our ontology (Fig. 1) were represented in *BioTriplex*, with variability in their frequency of occurrence. The largest number of mentions occurred for ‘increased expression’, with 210 mentions, and the smallest number of mentions occurred for ‘epigenetic marker’, with two mentions (Table 2).

**Table 2:** Frequency of occurrence of gene–disease relation type mentions (data shown are derived from Abstract, Introduction, Methods, Results, Discussion, and Conclusion sections, but not ‘Others’, consistent with Figs 4 and 5).

Ontology category	Relation type	Total mentions
	No relation	17
	Relation undefined	62
Disease causing	Pathological role	65
	Causative mutation	29
	Associated mutation	48
	Causative activation	4
	Causative inhibition	4
Disease modulating	Modulator increase disease	19
	Modulator decrease disease	5
	Genetic susceptibility	26
Gene expression	Increased expression	210
	Decreased expression	54
	Dysregulation	4
Diagnosis	Diagnostic tool	13
	biomarker	8
	Epigenetic marker	2
	Prognostic indicator	36
	Positive prognostic marker	8
	Negative prognostic marker	70
Therapy	Therapeutic target	47
	Therapy resistance	31

The most frequently represented disease classification group in linked triplets (755 occurrences) was of diseases dually-classified under the two EMBO Human Disease Ontology categories ‘disease of anatomical entity’ and ‘disease of cellular proliferation’, representing cancer subtypes. The second most frequently represented group (195 occurrences) represented diseases singly-classified under ‘disease of anatomical entity’, a more heterogeneous group that includes hypertension, multiple sclerosis and inflammatory bowel disease (Data 2, available as supplementary data at *Bioinformatics* online).

There were 312 different named genes included within linked triplets, with the frequency of occurrence ranging from one mention (131 different genes) to 38 mentions (gene GRAMD1A). The top three most frequently represented genes, GRAMD1A, CXCR4, and IDH1, have a variety of cellular functions but were all linked to mentions of different types of cancer, whilst the fourth most frequently represented gene, CXCL17, was linked to mentions of respiratory diseases including influenza, COVID-19, and tuberculosis (Table 3).

**Table 3:** Top 10 most frequently occurring human disease-linked genes in the corpus.

Gene	Previously-described cellular roles	Linked mentions	Diseases linked to gene in corpus
GRAMD1A	Cholesterol homeostasis	38	Hepatocellular carcinoma
CXCR4	Surface receptor with roles in signalling and cell migration	27	Hepatocellular carcinoma
IDH1	Enzyme involved in NADPH synthesis	27	Glioma, colorectal cancer, ovarian cancer, endometrial cancer
CXCL17	Chemoattractant for myeloid cells	25	Influenza, COVID-19, renal failure, tuberculosis, idiopathic pulmonary fibrosis, acute respiratory distress syndrome
PHD2 (EGLN1)	Cellular oxygen sensor	22	Pheochromocytoma, chronic myeloid leukaemia, polycythemia
P-gp (ABCB1)	Transports phospholipids and drugs across the cell membrane	22	Diabetes mellitus, leukaemia, pancreatic cancer, cancer
GPX3	Protects cells against oxidative damage	22	Lung fibrosis, sarcoidosis, chronic beryllium disease, asthma
BIRC5	Promotes proliferation and prevents apoptosis	19	Chronic obstructive airway disease, ovarian cancer, cancer
CXCR3	Surface receptor with roles in regulating T cells	18	Lymphoma; colon carcinoma; cancer, glycogen storage disease 1b
PD-1 (PDCD1)	Regulation of T cell functions	17	Lung adenocarcinoma, Hodgkin lymphoma, cancer

### 3.2 Machine learning

#### 3.2.1 Gene–disease RE

To evaluate the usefulness of the *BioTriplex* corpus as training data for the gene–disease RE task, we tested its potential to fine-tune the language model LLaMA 3.1 (Grattafiori *et al.* 2024) for extraction of gene–disease relation types from biomedical texts. We follow the standard definition of RE where the task is to classify the semantic relation between two pre-identified entity mentions within a given text segment. To establish baseline performance, a portfolio of benchmarks for zero-shot and few-shot RE was generated for both LLaMA 3.1 and two larger LLMs, GPT-4 (OpenAI *et al.* 2023) and Claude 3.7 Sonnet (Anthropic 2024). In addition, we evaluated BioBERT (Lee *et al.* 2020) and PubMedBERT (Gu *et al.* 2022) as baseline encoder-based models to provide a point of comparison with established transformer architectures.

We explored RE for both individual relation types (21 types) and general relation groups (five groups) (see Fig. 1). On average and across most relation types, 5-shot prompting outperformed the 0-shot variants (Tables 4–7). For individual relation types, fine-tuning the LLaMA 3.1 model with 8 billion parameters on the *BioTriplex* dataset yielded a micro-average *F*1 score of 0.63, representing an absolute improvement of 0.41 over the LLaMA 3.1 zero-shot model (*F*1 = 0.22) and 0.09 over the best-performing 5-shot prompted model (Claude 3.7, *F*1 = 0.54) (Table 4). Notably, the fine-tuned LLaMA 3.1 model (*F*1 = 0.63) also outperformed the fine-tuned BioBERT (*F*1 = 0.57), the strongest encoder-based baseline, by 0.06. In the general groups, the fine-tuned LLaMA 3.1 model achieved a micro-average *F*1 score of 0.68, representing an absolute improvement of 0.33 over the LLaMA 3.1 zero-shot model (*F*1 = 0.35) and 0.18 over the best-performing 5-shot model, GPT-4 (*F*1 = 0.50) (Table 6). The superior *F*1 scores of the fine-tuned LLaMA 3.1 models were attributable to substantial gains in precision (Tables 5 and 7). Across all baselines, the general relation groups yielded higher *F*1 scores than the individual relation types, suggesting that they are inherently easier to identify (Tables 4–7). We found that micro-average *F*1 scores scale while training on progressively larger subsets of *BioTriplex*, with performance trending upwards as dataset size increases (Data 7, available as supplementary data at *Bioinformatics* online).

**Table 4:** Individual relation-specific *F*1 scores for language models in gene–disease RE.

	Random baseline	BioBERT	PubMedBERT	GPT-4 zero-shot	GPT-4 few-shot	Claude 3.7 Sonnet zero-shot	Claude 3.7 Sonnet few-shot	LLaMA 3.1 8B zero-shot	LLaMA 3.1 8B few-shot	LLaMA 3.1 8B supervised
No relation	0.01	0	0	0	0	0	0	0.06	0	0.67
Relation undefined	0.2	0.07	0.27	0.08	0	0	0	0.05	0	0.19
Pathological role	0.15	0.49	0.24	0.41	0.28	0.28	0.34	0.18	0.26	0.65
Causative mutation	0.06	0.43	0.21	0.16	0.24	0.11	0.17	0.3	0.06	0.33
Associated mutation	0.2	0.81	0.39	0.12	0.16	0.54	0.65	0.2	0.32	0.5
Causative activation	0.01	0	0	0.09	0.4	0	1	0	0	0
Causative inhibition	0.01	0	0	0	0	0	0	0	0	0
Modulator decrease disease	0.02	0	0	0	0.33	0	0.5	0	0	0
Modulator increase disease	0.07	0.73	0.5	0	0	0.43	0.5	0	0	0.5
Genetic susceptibility	0.04	0.4	0.4	0.16	0.31	0.36	0.4	0.18	0.09	0.33
Increased expression	0.37	0.78	0.71	0.68	0.74	0.73	0.79	0.52	0.5	0.83
Decreased expression	0.15	0.55	0.67	0.48	0.57	0.65	0.79	0.32	0.5	0.76
Dysregulation	0.01	0	0	0.13	0.14	0	0.33	0.02	0.11	0
Diagnostic tool	0.04	0	1	1	1	0.67	0.8	0.1	0.62	0.62
Biomarker	0.03	0	0	0.22	0.29	0.1	0.6	0.04	0.06	1
Epigenetic marker	0.04	0	0	0.5	0.47	0.44	0.67	0.15	0.25	0.57
Prognostic indicator	0.11	0.63	0.71	0.39	0.34	0.37	0.37	0.27	0.21	0.59
Positive prognostic marker	0.04	0	0	0.42	0.57	0.8	0.89	0.18	0	0.89
Negative prognostic marker	0.15	0.47	0.48	0.54	0.68	0.62	0.62	0.41	0.26	0.7
Therapeutic target	0.05	0.37	0.18	0.18	0.23	0.2	0.29	0.1	0.29	0.91
Therapy resistance	0.06	0.8	0.37	0.52	0.48	0.52	0.46	0	0.42	0.62
Micro-average	0.1	0.57	0.47	0.35	0.4	0.43	0.54	0.22	0.26	0.63

**Table 5:** Micro-averaged metrics on individual relations for gene–disease RE on *BioTriplex*.

	Random baseline	BioBERT supervised	PubMedBERT supervised	GPT-4 zero-shot	GPT-4 few-shot	Claude 3.7 Sonnet zero-shot	Claude 3.7 Sonnet few-shot	LLaMA 3.1 8B zero-shot	LLaMA 3.1 8B few-shot	LLaMA 3.1 8B supervised
Precision	0.05	0.54	0.39	0.26	0.30	0.33	0.42	0.14	0.17	0.65
Recall	0.5	0.59	0.60	0.58	0.61	0.63	0.74	0.59	0.52	0.62
*F*I	0.1	0.57	0.47	0.35	0.40	0.43	0.54	0.22	0.26	0.63

**Table 6:** Relation group-specific *F*1 scores by model for the gene–disease RE task.

	Random baseline	BioBERT	PubMedBERT	GPT-4 zero-shot	GPT-4 few-shot	Claude 3.7 Sonnet zero-shot	Claude 3.7 Sonnet few-shot	LLaMA 3.1 8B zero-shot	LLaMA 3.1 8B few-shot	LLaMA 3.1 8B supervised	Positive samples
No relation	0.01	0	0	0	0	0	0	0.04	0.05	0	1
Relation undefined	0.2	0.11	0	0.07	0	0	0.06	0.18	0.07	0.29	23
Disease causing	0.33	0.71	0.67	0.48	0.67	0.49	0.51	0.39	0.48	0.71	45
Disease modulating	0.12	0.36	0.28	0.17	0.18	0.24	0.24	0.13	0.15	0.52	13
Gene expression	0.44	0.71	0.62	0.58	0.71	0.65	0.72	0.57	0.58	0.81	72
Diagnosis	0.3	0.59	0.61	0.55	0.68	0.52	0.66	0.44	0.49	0.76	40
Therapy	0.11	0.32	0.27	0.3	0.38	0.37	0.28	0.29	0.3	0.56	11
Micro average	0.24	0.59	0.49	0.41	0.5	0.45	0.49	0.35	0.41	0.68	

**Table 7:** Micro-averaged metrics on general relation groups for gene–disease RE on *BioTriplex*.

	Random baseline	BioBERT supervised	PubMedBERT supervised	GPT-4 zero-shot	GPT-4 few-shot	Claude 3.7 Sonnet zero-shot	Claude 3.7 Sonnet few-shot	LLaMA 3.1 8B zero-shot	LLaMA 3.1 8B few-shot	LLaMA 3.1 8B supervised
Precision	0.16	0.69	0.63	0.31	0.38	0.38	0.41	0.23	0.30	0.69
Recall	0.5	0.51	0.40	0.61	0.72	0.57	0.60	0.71	0.66	0.67
*F*I	0.24	0.59	0.49	0.41	0.50	0.45	0.49	0.35	0.41	0.68

To further evaluate the robustness of our RE baselines, we also explored fine-tuning of the LLaMA 3.1 model on the gene–disease relations of the previously-described BioRED dataset (Luo *et al.* 2022). We compared the performance of LLaMA 3.1 zero-shot, few-shot, and fine-tuned models with the pre-LLM state-of-the-art *BioREx* model (Lai *et al.* 2025). The fine-tuned LLaMA 3.1 model performed most strongly, achieving a micro-averaged *F*1 score of 0.76 and surpassing the *BioREx F*1 score of 0.71 by 0.06 (Data 4, available as supplementary data at *Bioinformatics* online), indicating good generalization to related biomedical tasks. Because BioRED defines only broad relation categories while BioTriplex contains 21 fine-grained subtypes, absolute performance differences across datasets are expected. This competitive performance of LLaMA 3.1 against an established biomedical RE benchmark confirms it as an appropriate model for evaluating the usefulness of *BioTriplex* as training data.

#### 3.2.2 NER of genes, diseases, and relations

To explore the potential of *BioTriplex* to support the training of language models for NER tasks, we used it to fine-tune LLaMA 3.1 for NER of the *BioTriplex* genes, diseases, and relations entity groups. LLMs typically perform relatively poorly in supervised structured prediction tasks (Keloth *et al.* 2024, del Moral-González et al. 2025, Nagar *et al.* 2025). Bidirectional transformers are better suited for such tasks due to their optimization for contextual token-level representations. We therefore created two different bidirectional transformer baselines using (i) BioBERT (Lee *et al.* 2020) and (ii) PubMedBERT (Gu *et al.* 2020) within the DyGIE++ framework (Wadden *et al.* 2019). For LLaMA 3.1, we experimented with two prompting approaches: (i) instructing the LLM to generate a JSON-formatted list of entity spans and types (NER-JSON) and (ii) prompting it to output the sequence of words in the text paired with its corresponding entity type tag (NER-Sequence) (Fig. 3), each within zero-shot, few-shot, and supervised learning scenarios. All fine-tuned models exceeded the performance of the baseline, PubTator 3.0 (Wei *et al.* 2024), in the gene and disease categories (PubTator Relation types are derived from BioRED and therefore are not directly comparable with *BioTriplex*). Both bidirectional transformer models substantially outperformed LLaMA 3.1 in zero-shot and few-shot scenarios, with the best-performing model being PubMedBERT-based Dyige++, despite it having significantly fewer parameters than LLaMA 3.1 (110 million vs. 8 billion). The supervised LLaMA 3.1 models, however, showed substantial improvements in performance, achieving *F*1 scores that approached or equalled those of the bidirectional transformer models (Table 8). The LLaMA 3.1 NER-Sequence prompting approach outperformed the LLaMA 3.1 JSON-based format, suggesting that sequence tagging may offer more stable grounding for entity recognition in generative models. Across all models, NER of relation types proved more challenging than the identification of genes or diseases (Table 8).

**Table 8:** NER precision, recall, and *F*1 scores for diseases, genes, and relations.

	PubTator 3.0	BioBERT	Dygie++ (PubMedBERT base)	LLaMA 3.1 8B (NER-SEQ) zero-shot	LLaMA 3.1 8B (NER-SEQ) few-shot	LLaMA 3.1 8B (NER-SEQ) supervised	LLaMA 3.1 8B (NER-JSON) zero-shot	LLaMA 3.1 8B (NER-JSON) few-shot	LLaMA 3.1 8B (NER-JSON) supervised
Diseases									
Precision	0.38	0.76	0.79	0.30	0.51	0.77	0.34	0.30	0.77
Recall	0.95	0.92	0.91	0.45	0.46	0.85	0.22	0.25	0.76
*F*I	0.55	0.89	0.85	0.36	0.49	0.81	0.26	0.27	0.77
Genes									
Precision	0.63	0.85	0.90	0.34	0.58	0.92	0.53	0.59	0.84
Recall	0.71	0.94	0.96	0.24	0.26	0.87	0.21	0.24	0.82
*F*I	0.70	0.80	0.90	0.30	0.40	0.90	0.30	0.30	0.80
Relations									
Precision	N/A	0.27	0.60	0.01	0.03	0.48	0.01	0.01	0.26
Recall	N/A	0.42	0.38	0.04	0.20	0.20	0.08	0.14	0.08
*F*I	N/A	0.32	0.47	0.02	0.06	0.28	0.01	0.02	0.13

## 4 Discussion

*BioTriplex* is a new resource that can support the training of language models to extract and interpret the complex relationships described between genes and diseases in research literature. By creating our corpus from full-text research papers, we were able to include a greater diversity of information than previously-described corpora based on abstracts only (Figs. 4 and 5; Data 5, available as supplementary data at *Bioinformatics* online). Training models on diverse datasets can increase generalizability, even when the quantity of data is relatively small (Galea *et al.* 2018, Luo *et al.* 2023). In the biomedical domain, limiting training data to study abstracts risks excluding important details. For example, in reports of drug trials, harmful effects described in the full text of an article are often omitted from the abstract (Bernal-Delgado and Fisher 2008) and in studies of the effect of environmental factors on cancer incidence, the full range of variables and outcomes is often only described in the full text (Duyx *et al.* 2019). In *BioTriplex*, we found that Discussion sections contained the richest source of information relevant to disease–gene relationships (Fig. 4). Together, these findings suggest a risk of reporting bias when models are trained on abstracts alone and underline the need for developing scalable methodology for full-text articles.

Useful language models need the ability to explain the significance of extracted inter-entity relationships to different research questions. We identified 21 different gene–disease relation types and built a representative ontology which organizes them into five general categories: ‘disease causing’, ‘disease modulating’, ‘gene expression’, ‘diagnosis’, and ‘therapy’ (Fig. 1). The granularity of relation type labelling in *Biotriplex* is designed to allow more specific and precise fine-tuning of language models for gene–disease RE tasks. For example, a researcher interested in the genetic basis of a specific disease might wish to not only extract mentions of linked gene mutations but also to distinguish between those with demonstrated causative roles (‘causative mutations’ in our ontology) and those that are merely associated, with no causative roles reported (‘associated mutations’ in our ontology).

We evaluated *BioTriplex* by using it to fine-tune the open-source language model, LLaMA 3.1 (Grattafiori *et al.* 2024), for gene–disease RE. To create strong baselines, we also measured the zero-shot and few-shot performance of both LLaMA 3.1 and two larger LLMs, GPT-4 and Claude 3.7 Sonnet. The supervised LLaMA 3.1 model surpassed both supervised encoder-based baselines (BioBERT and PubMedBERT) achieving higher overall *F*1 scores than either BERT variant. Notably, the supervised LLaMA 3.1 model also outperformed all zero- and few-shot prompting methods in the majority of individual relation types, affirming the value of task-specific fine-tuning. Even with the rare *epigenetic marker* relation type, where no examples exist in the training or validation sets, the supervised model performed above random, indicating some degree of generalization. On average, 5-shot prompted models consistently outperformed their 0-shot counterparts in terms of precision, and supervised fine-tuning further amplified this effect. This was attributable to a tendency for the zero- and few-shot models to over-predict relations in *BioTriplex*, assigning more labels per instance than the supervised variant. In contrast, the fine-tuned LLaMA 3.1 model achieved better precision by producing more selective predictions, albeit with a slight reduction in recall, and yielding a net gain in *F*1 score. Overall, these findings demonstrate that *BioTriplex* supports meaningful performance improvements in fully supervised learning scenarios and can lead to refinements in the factual precision of model outputs (Tables 4–7).

We also explored the potential of the *BioTriplex* dataset for fine-tuning LLaMA 3.1 for NER of the *Biotriplex* gene, disease, and relation entities. In the absence of fine-tuning, the performance of our two different baseline LLaMA 3.1 NER models lagged behind two previously-described bidirectional transformer NER models, despite their larger parameter size. This observation aligns with prior findings that encoder-only architectures are better suited to structured prediction tasks (Keloth *et al.* 2024, del Moral-González *et al.* 2025, Nagar et al. 2025). Autoregressive LLMs such as LLaMA 3.1 generate text by predicting the next token in a left-to-right manner and are therefore better suited to generation tasks. Notably, however, fine-tuning of LLaMA 3.1 on *BioTriplex* resulted in improved NER scores that approached or equalled those achieved using bidirectional transformers (Table 8).

A typical biomedical research paper includes both novel findings and relevant background, and we show that most paper subsections contain both types of information (Figure 5). The annotation of *BioTriplex* entities with labels that distinguish between novel and background entities could support the training of future models that can pinpoint those concepts that are actively studied (as similarly demonstrated for pathogens in Yepes *et al.* (2021)), increasing the trustworthiness of output predictions.

In conclusion, our study supports the development of efficient data extraction methods that can better capture the nuances of biomedical texts. In *BioTriplex*, we contribute a new, performance-validated corpus to a domain that is under-resourced in full-text corpora. We demonstrate that a relatively small, but diverse and comprehensively labelled dataset can be used to fine-tune an LLM for specialized biomedical tasks, outperforming other language models and increasing the factual precision and real-world usefulness of data outputs.

## Supplementary Material

btag037_Supplementary_Data

## Data Availability

The BioTriplex corpus and the data and code underlying this article are available at https://github.com/PanagiotisFytas/BioTriplex.
