# Supplementary material for: BioTriplex: a full-text annotated corpus for fine-tuning language models in gene-disease relation extraction tasks
Source: Bioinformatics. 2026 Jan 21;42(2):btag037. doi: 10.1093/bioinformatics/btag037 (PMC12883087; doi:10.1093/bioinformatics/btag037)

Supplementary data 1.

Guidelines for annotating PubMed Articles for *BioTriplex* project using Multi-document Annotation Environment (MAE)

Author: Charlotte Collins

1.1 Overview

The aim of the task is to annotate texts by marking words and phrases ('entities') that relate to human diseases, genes and the relationships between them. Markable entities correspond to one of three defined categories: 'Human Diseases', 'Genes', and 'Relations'. Each marked entity is labelled with a listed term or terms ('assertions'). The associations between the different categories of entity are also labelled.

1.2 Annotation Tool

The texts are annotated using Multi-Annotation Environment (MAE) software http://keighrim.github.io/mae-annotation/. Annotation tasks in MAE are defined using a DTD file that is specific to the task (see Appendix). MAE requires input files in .txt format and creates a .xml file output for each annotated text.

1.3 Source texts

The source texts are CC BY-licenced biomedical journal full length papers obtained from the PubMed Central Open Access database. Titles, authors, affiliations, keywords and other extraneous text are removed. Separate .txt files are created for each section of each paper e.g. Abstract, Introduction, Results, Materials and Methods, Discussion and Conclusions. Some papers will contain other additional sections e.g. 'Summary' or 'Case notes'; other papers may lack one or more sections.

2.1 Marking and labelling entities in text

An entity is identified within a source text and marked as either a 'Human Disease', a 'Gene', or a 'Relation'. Each marked entity is then labelled with one or more assertion terms that provide information. Explanations of these terms are shown in **Table 1** and examples are shown in **Table 2**.

In this project we will be annotating articles that explore the roles of genes in human diseases.

- **Human diseases** are any disease listed in the EMBL-EBI Human Disease Ontology (https://www.ebi.ac.uk/ols/ontologies/doid). The type of disease is labelled using defined ontology terms.
- **Genes** are human gene names, symbols or synonyms, using https://www.genecards.org as a reference.
- **Relations** are the type of relationship reported to occur between a gene and a human disease.

Category	Assertion Type 1	Assertion Type 2
**Human Disease**	*Location* (1 label)<br>background; result	*Disease type* (1 or 2 labels)<br>disease by infectious agent; disease of anatomical entity; disease of cellular proliferation; disease of mental health; disease of metabolism; genetic disease; physical disorder; syndrome
**Gene**	*Location* (1 label)<br>background; result	n/a
**Relation**	*Location* (1 label)<br>background; result	*Relation type* (1 label)<br>no relation; relation undefined; pathological role; causative activation; causative inhibition; causative mutation; modulator decrease disease; modulator increase disease; biomarker; associated mutation; dysregulation; increased expression; decreased expression; epigenetic marker; therapy resistance; prognostic indicator; negative prognostic marker; positive prognostic marker; therapeutic target; diagnostic tool; genetic susceptibility

Table 1. Categories of markable entity and assertions used to label them.

Markable Entity	Category	Assertion Type 1	Assertion Type 2
		Location	***Disease type(s)***
lung adenocarcinoma	Human Disease	background/result	disease of anatomical location; disease of cellular proliferation.
atherosclerotic cardiovascular disease	Human Disease	background/result	disease of anatomical entity; -
Loeys-dietz syndrome	Human Disease	background/result	genetic disease; syndrome
		Location	***n/a***
SLC02A1	Gene	background/result	n/a
PCSK5	Gene	background/result	n/a
Angiotensin I converting enzyme	Gene	background/result	n/a
		Location	***Relation type***
highly expressed	Relation	background/result	increased expression
drug resistance	Relation	background/result	therapy resistance
causative mutation	Relation	background/result	causative mutation

Table 2. Examples of markable entities.

2.2 Guidelines for marking and labelling entities

(a) General points

(i) Mark every example of each Gene and Human Disease which occurs in the text.

(ii) Mark every example of each Relation which describes the relationship between a marked Gene and a marked Human Disease.

(iii) Mark the whole word or phrase and do not include any blank space or punctuation (e.g. brackets) outside the boundaries.

(iv) In a few cases, the spans of entities may overlap with others.

(v) Do not mark extraneous text. Mark multi-span entities (entities composed of two or more text segments) where necessary.

(b) Human Diseases

(i) Mark every example of a Disease name that corresponds to a disease listed in the Human Disease Ontology database.

(ii) Mark abbreviations of listed Diseases (abbreviations are not themselves listed in the Human Disease ontology).

(iii) Disease abbreviations and long forms should be annotated as separate entities. For example, in the phrase ‘Duchenne Muscular Dystrophy (DMD)’, ‘Duchenne Muscular Dystrophy’ and ‘DMD’ should each be marked as a separate entity and will each correspond to the same human disease identifier.

(c) Genes

(i) Mark every example of a human gene (symbol, name or synonym) that corresponds to a gene listed in the Genecard database. Do not mark genes stated to be that of any other non-human species.

(ii) Do not mark incomplete names of genes or the names of gene families e.g. mark ‘WNT1’; do not mark ‘Wnt family genes’.

(iii) Do not mark genes that are named as part of the name or description of a biochemical mechanism or pathway e.g. in ‘Wnt/Beta-catenin pathway’ do not mark ‘Beta-catenin’.

(d) Relations

(i) Mark Relations (words and phrases) that both (a) correspond to the Relation types listed in Table 1 and Figure 1 and (b) describe the relationship between a marked Gene and a marked Human Disease.

(ii) Words and phrases that have different surface forms but which correspond to the sense of the listed Relation types should be marked. Words or phrases which describe relationships but which do not correspond to the sense of a listed Relation type should not be marked.

(iii) Do not mark words or noun phrases that correspond to a listed Relation type but which describe the relationship between any marked entities other than a marked Gene and a marked Disease, e.g. do not mark the relationship between a Gene and another Gene.

(iv) Text which explicitly reports the absence of a relationship between a marked Gene and a marked Disease should be labelled with the Relation type ‘No relation’. Text which reports a relationship, but the nature of that relationship is unspecified, should be labelled with the Relation type ‘Relation undefined’.

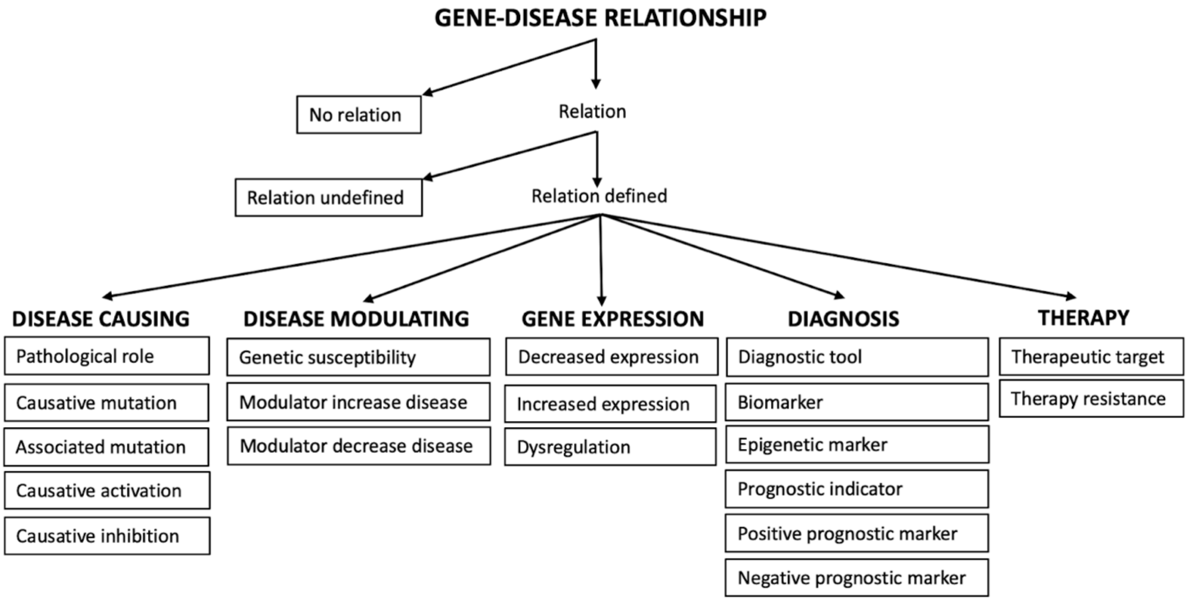

Figure 1. Ontology of Gene-Human Disease Relation types occurring in texts. Terms within boxes represent the categories of annotated relation type.

2.3 Guidelines for labelling entities with assertions

(a) Each marked entity should be labelled with assertion term(s) as shown in in Tables 1 and 2.

(b) All entities should be labelled with a *Location* assertion. Entities which form part of the background information (e.g. previous work) should be labelled with the assertion ‘background’. Entities which form part of the description or discussion of new

experimental results, including all methodology, should be labelled with the assertion 'result'. All sections of experimental papers may include background information and therefore the section title cannot be used to determine the appropriate assertion to use.

(c) Genes are labelled only with a *Location* assertion.

(d) Human Diseases are additionally labelled with 1 or 2 *Disease type* assertions that describe the type of disease as classified in the Human Disease Ontology (Table 1). Refer to the Human Disease Ontology to determine the correct assertion(s) to use. Where only 1 assertion is used for labelling, select '-' in the unlabelled column.

(e) Relations are additionally labelled with a *Relation type* assertion as described in Table 1 and labelled in red in Figure 1. A Gene and a Human Disease that are explicitly stated to have no relationship should be labelled with the assertion 'no relation'. Where they are stated to have a relationship, but the relationship is not defined, label with the assertion 'Relation undefined'. All other Relations should be labelled with an assertion that closely corresponds to their sense, though surface forms may often vary from the terms listed.

2.4 Worked examples of marking entities in text

Genes are highlighted in Green; Human Diseases are highlighted in Blue; Relations are highlighted in Yellow.

(i) *Our finding suggested SETD2 as a potential epigenetic marker in LUAD patients*

In this example, all marked entities are labelled with the Location assertion 'Result'.
'*Epigenetic marker*' is additionally labelled with the Relation type assertion 'Epigenetic marker'.
'*LUAD*' (an abbreviation of *Lung adenocarcinoma*) is additionally labelled with the Disease type assertions 'Disease of anatomical entity' and 'Disease of cellular proliferation'.

(ii) *CDKN2BAS1/ANRIL , located in the 9p21 chromosomic region, has been reported in numerous studies as a genetic risk locus for CAD, intracranial aneurysms and diverse cardiometabolic disorders*

In this example, all marked entities are labelled with the Location assertion 'Background'.
The two alternative gene names (which correspond to the same locus) are marked separately.
'*Genetic risk locus*' is additionally labelled with the Relation type assertion 'Genetic susceptibility'.
'*CAD*' (an abbreviation of Coronary Artery Disease) is additionally labelled with the Disease type assertion 'Disease of anatomical entity'.

‘Intracranial aneurysms’ is additionally labelled with the Disease type assertion ‘Disease of anatomical entity’.

‘*Cardiometabolic disorders*’ is not marked because this is a general term that refers to a related group of disorders, not the name of a specific human disease.

(iii) *Duchenne muscular dystrophy (DMD) is an X-linked inherited neuromuscular disorder due to mutations in the dystrophin gene*

In this example, all marked entities are labelled with the Location assertion ‘Background’.
The full name of the human disease, ‘*Duchenne muscular dystrophy*’ and its abbreviation, ‘*DMD*’ are marked separately. Each corresponds to the same identifier in the Human Disease Ontology Database and is labelled with the Disease type assertions ‘Disease of anatomical entity’ and ‘Genetic disease’.
‘*Due to mutations*’ is labelled with the Relation type assertion ‘Causative mutation’.
The gene name ‘*dystrophin*’ corresponds to the human *DMD* gene in the Gene Ontology Database.

(iv) *activation of Wnt signalling is nonetheless thought to play an important role in breast tumorigenesis*

This is an example of a text segment which does not contain any markable entities.
‘*Wnt*’ is not marked because it describes a signalling pathway and is not the name of an individual human gene.
‘*Breast tumourigenesis*’ is not marked because it describes a pathological process and is not the name of a human disease.
‘*Role*’ is not marked because in this text, it does not describe the relationship between two marked entities.

3.1 Entity linking (marking the relationships between entities)

MAE can be used to record the relationships between marked entities. In this project we are marking the links between groups of three marked entities, each set comprising one Human disease, one Gene and one Relation.

3.2 General guidelines for entity linking

(a) The linked entities are typically derived from he same sentence but may come from different sentences within the text.

(b) Only link entities between which there is a clear relationship.

(c) Create links of a single Human Disease with a single Gene and a single Relation.

(d) Do not link incomplete groups of fewer than three entities.

(e) Where one Gene relates to more than one Human Disease and/or more than one Relation, or one Human Disease relates to more than one Gene and/or more than one Relation, create several linked groups.

(f) Some marked entities may be included in more than one linked group.

(g) Some marked entities cannot be reasonably linked within a group of three marked entities. Leave them non-linked.

3.3 Worked example of entity linking

high expression of METTL3, HNRNPA2B1, and YTHDF3 were related to the poor prognosis of osteosarcoma

This text segment contains six separate groups of related entities that can be linked together.

(i) Link Gene: *METTL3* and Relation: *high expression* and Human Disease: *osteosarcoma*
(ii) Link Gene: *HNRNPA2B1* and Relation: *high expression* and Human Disease: *osteosarcoma*
(iii) Link Gene: *YTHDF3* and Relation: *high expression* and Human Disease: *osteosarcoma*
(iv) Link Gene: *METTL3* and Relation: *poor prognosis* and Human Disease: *osteosarcoma*
(v) Link Gene: *HNRNPA2B1* and Relation: *poor prognosis* and Human Disease: *osteosarcoma*
(vi) Link Gene: *YTHDF3* and Relation: *poor prognosis* and Human Disease: *osteosarcoma*

4.1 Workflow for annotation of files using MAE

(a) Open MAE.

(b) Go to 'File' and select 'New Task Definition'. Load a .txt file of the DTD (see Appendix) to set up the annotation task. There will now be three coloured tabs labelled 'GENE', 'DISEASE' and 'RELATION' and a non-coloured tab labelled 'ENTITY_LINKING'.

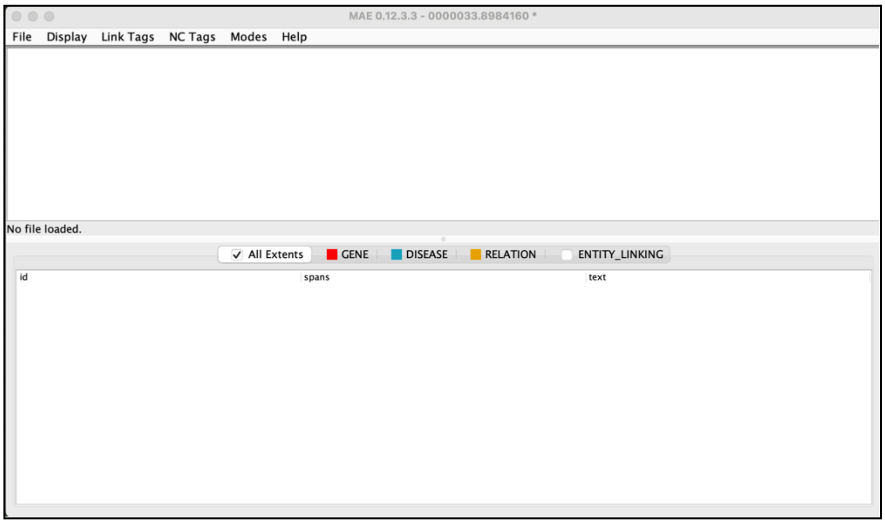

(c) Open a text file. MAE converts .txt files to .xml format. The screen should now look like this:

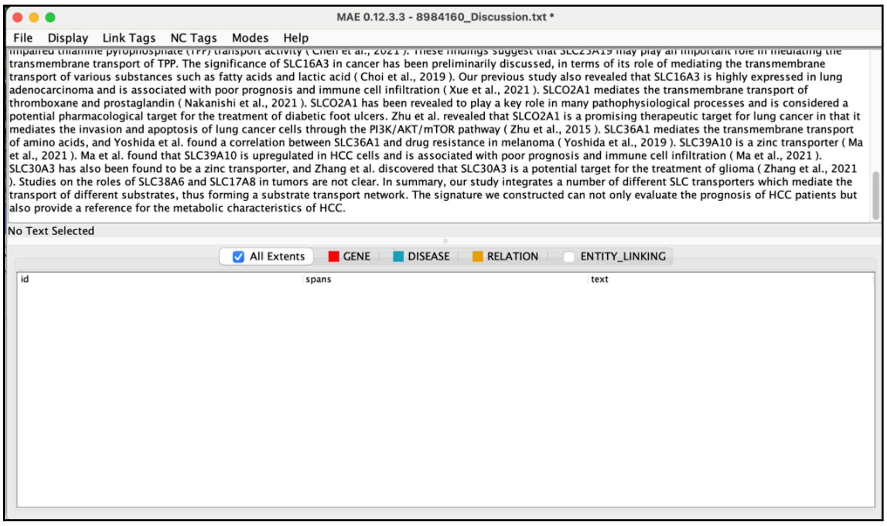

(d) To mark an entity in the text, create an Extent Tag. Highlight the word or phrase and R click (or R + L depending on your settings). Click on 'Create Extent Tag with selected text' to bring up a menu. Select either 'DISEASE', 'GENE' or 'RELATION' to create the appropriate Extent tag.

The Extent tag ID, spans and marked text are now recorded under the appropriate tab (DISEASE in the example shown). The marked text remains highlighted in a colour that corresponds to the type of Extent tag.

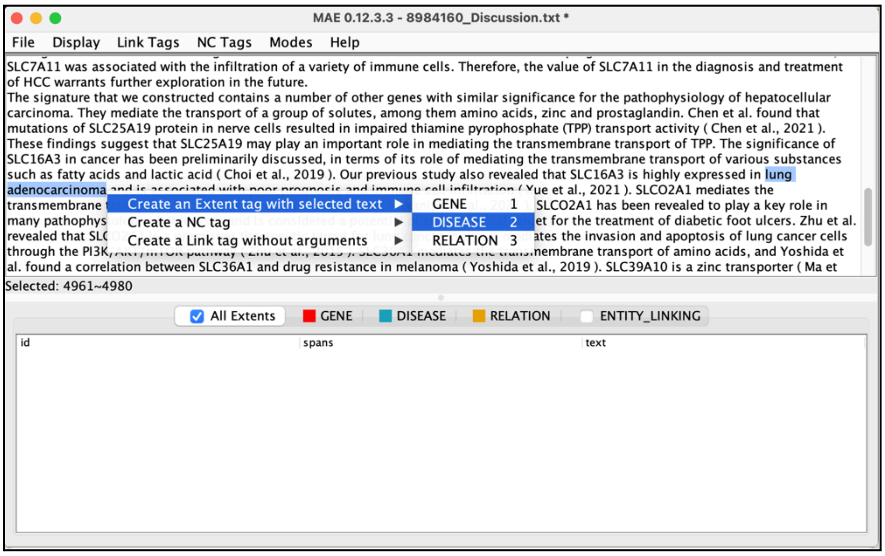

(e) Label each marked entity by selecting the appropriate assertion(s) from the dropdown box(es) on the right of the screen.

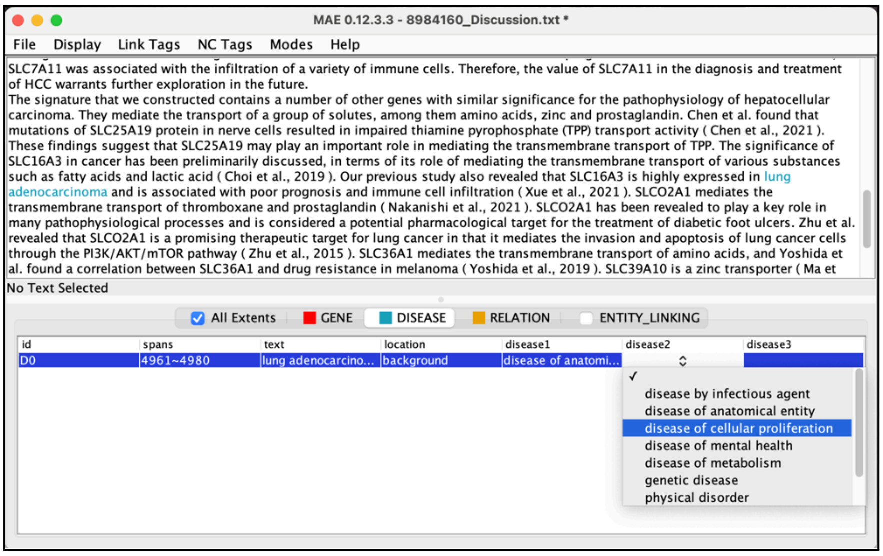

(f) Tag and label all other markable entities in the text. This will create a list under each of the ‘DISEASE’, ‘GENE’ and ‘RELATION’ tabs and a collated list under the ‘All Extents’ tab.

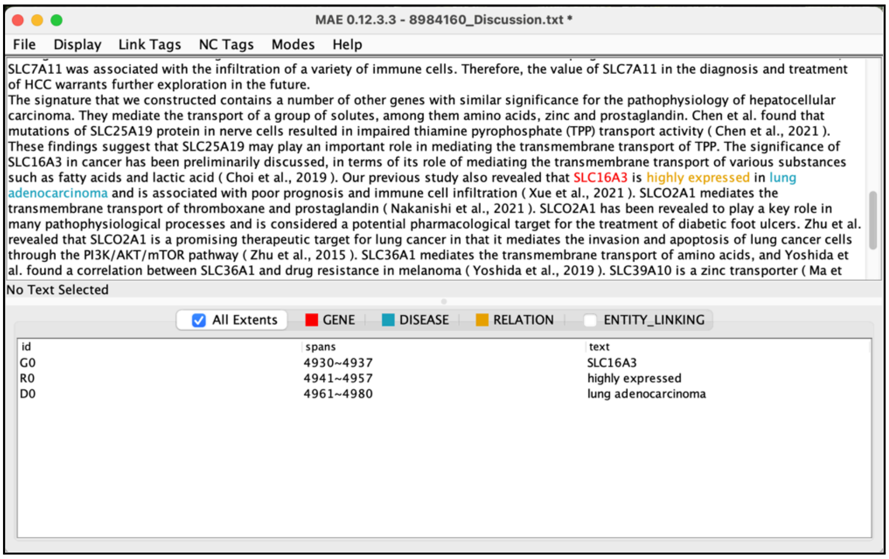

(g) To mark a link between a group of three related entities, create a Link Tag. Note that for illustrative purposes, in this example only the three relevant entities are highlighted. Click (R or R+L) on any blank space in the document to bring up a dropdown menu. Select 'Create Link Tag with no arguments associated' and then select 'ENTITY_LINKING'.

(h) An empty row will appear under the 'ENTITY_LINKING' tab. Highlight part or all of the first marked entity that you want to link. Click (R or R+L) to bring up a dropdown menu and select 'Set '*id* (*text*)' as argument of link tag'.

(i) A window will appear. Use the dropdown menu to 'Select Argument Type' ('gene' in the example shown) and the Link Tag id (E0 in the example shown).

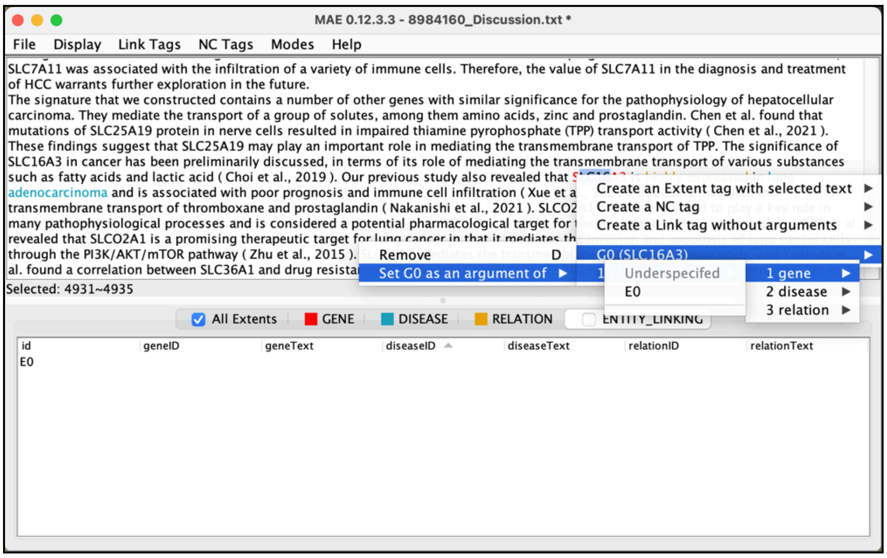

(j) The ID and text of the marked entity will appear under ‘disease’, ‘gene’ or ‘relation’ in the ENTITY_LINKING tab. Repeat the process to add the other two entities to be linked. There should now be one entry in each of the ‘disease’, ‘gene’ and ‘relation’ categories.

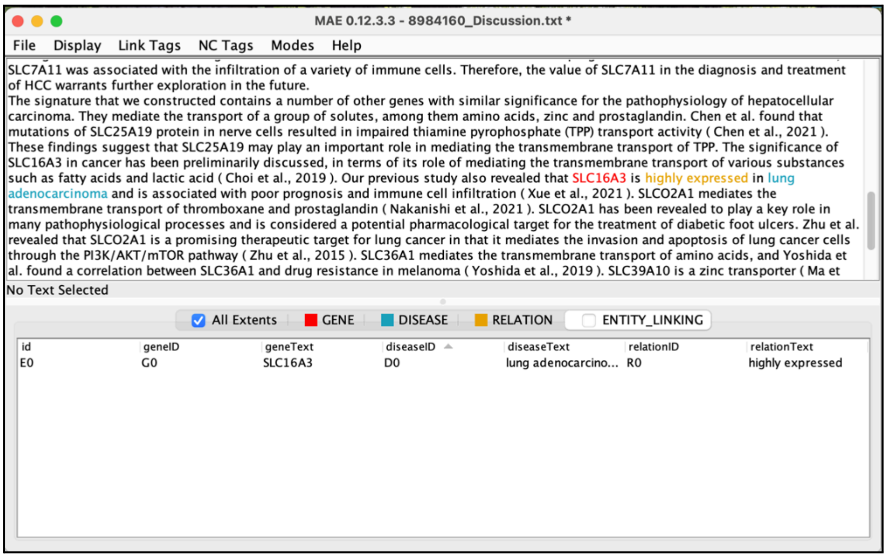

(k) Create new ENTITY_LINKING tags and repeat the process to mark the links between any other groups of entities. This will create a list under the ENTITY_LINKING tab.

(l) Save and export the completed annotation as an XML file.

APPENDIX TO ANNOTATION SCHEME

DTD used to define annotation parameters in MAE

```
<!--
  ~ KEY TERMS
  ~ A DTD is a Document Type Definition.
  ~ It defines the structure and the legal elements and attributes of
an XML document.
  ~ Main building blocks are elements, attributes, entities, PCDATA,
and CDATA.
-->

<!ENTITY name "Genomics_ConceptTask" >

<!-- PCDATA is parsed character data for tool to treat as markup-->
<!ELEMENT GENE ( #PCDATA ) >
<!ELEMENT DISEASE ( #PCDATA ) >
<!ELEMENT RELATION ( #PCDATA ) >
<!ELEMENT ENTITY_LINKING EMPTY >
<!ATTLIST ENTITY_LINKING arg0 IDREF prefix="gene" #REQUIRED>
<!ATTLIST ENTITY_LINKING arg1 IDREF prefix="disease" #REQUIRED>
<!ATTLIST ENTITY_LINKING arg2 IDREF prefix="relation" #REQUIRED>

<!-- fixed value set with a default value; IMPLIED means attribute is
optional-->
<!ATTLIST GENE location ( background | result )"-" #IMPLIED >
<!ATTLIST RELATION location ( background | result )"-" #IMPLIED >
<!ATTLIST RELATION relation type (no relation | relation undefined |
causative activation | causative inhibition | causative mutation |
modulator decrease disease | modulator increase disease | biomarker |
associated mutation | increased expression | decreased expression |
epigenetic marker | therapy resistance | prognostic indicator |
negative prognostic marker | positive prognostic marker | therapeutic
target | diagnostic tool | genetic susceptibility )"-" #IMPLIED >
<!ATTLIST DISEASE location ( background | result )"-" #IMPLIED >
<!ATTLIST DISEASE disease1 ( disease by infectious agent | disease of
anatomical entity | disease of cellular proliferation | disease of
mental health | disease of metabolism | genetic disease | physical
disorder | syndrome )"-" #IMPLIED >
<!ATTLIST DISEASE disease2 ( disease by infectious agent | disease of
anatomical entity | disease of cellular proliferation | disease of
mental health | disease of metabolism | genetic disease | physical
disorder | syndrome | - )"-" #IMPLIED >
```

Supplementary data 2. Frequency of linked Disease-Gene-Relation triplets associated with different disease group categories. Some diseases are classified under one EMBO Human Disease Ontology category and others are classified under two categories.

EMBO Human Disease Ontology categories	Linked Disease-Gene-Relation triplets in corpus
disease by infectious agent	3
disease of anatomical entity	195
disease of cellular proliferation	72
disease of mental health	45
disease of metabolism	3
disease by infectious agent, disease of anatomical entity	22
disease of anatomical entity, disease of cellular proliferation	755
disease of anatomical entity, disease of metabolism	7
disease of anatomical entity, genetic disease	40
disease of cellular proliferation, genetic disease	1

Supplementary data 3. Distribution % (counts) by entity category and relation type across dataset splits.

	Overall	Training Set	Test Set	Validation Set
Entity Category				
Genes	65.44% (14881)	63.49% (9919)	71.27% (2890)	67.69% (2072)
Relations	3.38% (769)	3.41% (532)	3.53% (143)	3.07% (94)
Diseases	31.18% (7089)	33.11% (5172)	25.20% (1022)	29.24% (895)
Relations by Type				
no relation	2.21% (17)	2.82% (15)	0.70% (1)	1.06% (1)
relation undefined	8.19% (63)	6.58% (35)	13.29% (19)	9.57% (9)
pathological role	8.45% (65)	8.65% (46)	6.99% (10)	9.57% (9)
causative mutation	3.77% (29)	3.20% (17)	2.80% (4)	8.51% (8)
associated mutation	6.63% (51)	6.58% (35)	9.09% (13)	3.19% (3)
causative activation	0.52% (4)	0.38% (2)	0.70% (1)	1.06% (1)
causative inhibition	0.52% (4)	0.56% (3)	0.70% (1)	0.00% (0)
modulator increase disease	2.47% (19)	3.01% (16)	1.40% (2)	1.06% (1)
modulator decrease disease	0.65% (5)	0.56% (3)	1.40% (2)	0.00% (0)
genetic susceptibility	3.38% (26)	4.32% (23)	2.10% (3)	0.00% (0)
increased expression	27.70% (213)	26.50% (141)	29.37% (42)	31.91% (30)
decreased expression	7.28% (56)	6.95% (37)	9.09% (13)	6.38% (6)
dysregulation	0.52% (4)	0.19% (1)	0.70% (1)	2.13% (2)
diagnostic tool	1.69% (13)	1.50% (8)	0.70% (1)	4.26% (4)
biomarker	1.04% (8)	1.13% (6)	1.40% (2)	0.00% (0)
epigenetic marker	0.26% (2)	0.00% (0)	1.40% (2)	0.00% (0)
prognostic indicator	4.68% (36)	5.45% (29)	2.80% (4)	3.19% (3)
positive prognostic marker	0.91% (7)	0.56% (3)	2.80% (4)	0.00% (0)
negative prognostic marker	8.97% (69)	10.34% (55)	6.29% (9)	5.32% (5)
therapeutic target	6.11% (47)	6.77% (36)	2.80% (4)	7.45% (7)
therapy resistance	4.03% (31)	3.95% (21)	3.50% (5)	5.32% (5)
Total Relations	100.00% (769)	100.00% (532)	100.00% (143)	100.00% (94)

Supplementary data 4. Micro-averaged F1 metrics for RE on the gene-disease <G, D> relations of BioRed.

Model	F1
Random Baseline	0.25
LLaMA 3.1 Zero-shot	0.34
LLaMA 3.1 Few-shot	0.38
LLaMA 3.1 Supervised	0.76
BioRex <G,D> pairs Supervised	0.71

***Supplementary data 5*.** Comparison of biomedical corpora manually annotated with diseases and genes.

	Corpus size	Annotation method	Entity types	Total entities	Task(s)
BioTriplex	100 full texts (604 subsections)	manual	Genes; Human Diseases; Gene-Disease Relation Types	22,970	NER; Relation extraction
BioRED (Luo et al., 2022)	600 abstracts	automatic/manual	Gene; Disease; Chemical; Variant; Species; Cell line	20,419	NER; Relation extraction
RENET2 (Su et al., 2021)	1000 abstracts	500 manual and 500 automatic/manual	Genes; Diseases	2,383 in manual subset	Relation extraction
NCBI disease corpus (Dogan et al., 2014)	793 abstracts	automatic/manual	Diseases	6,892	NER
AGAC (Wang et al., 2019)	500 abstracts	manual	Genes; Diseases; Proteins; Enzymes	5,741	NER; Thematic relation identification
EU-ADR (van Mulligen et al., 2012)	100 abstracts	manual	Genes; Disorders; Drugs; Targets	7,011	NER; Relation extraction

Supplementary data 6. Variability of different relation types spans per *BioTriplex* split.

Relation type	Split	Instances	Unique Expressions	Mean Tokens/mention
associated mutation	test	13	9	1.923 ± 0.730
	train	35	25	2.286 ± 1.232
	val	3	2	1.667 ± 0.943
biomarker	test	2	1	1.000 ± 0.000
	train	6	4	1.167 ± 0.373
	val	0	0	0.000 ± 0.000
causative activation	test	1	1	4.000 ± 0.000
	train	2	2	3.000 ± 1.000
	val	1	1	3.000 ± 0.000
causative inhibition	test	1	1	8.000 ± 0.000
	train	3	3	4.667 ± 1.700
	val	0	0	0.000 ± 0.000
causative mutation	test	4	4	1.750 ± 0.829
	train	17	12	3.176 ± 1.504
	val	8	7	3.500 ± 1.225
decreased expression	test	13	10	2.154 ± 1.099
	train	37	24	2.054 ± 1.064
	val	6	4	1.833 ± 1.067
diagnostic tool	test	1	1	1.000 ± 0.000
	train	8	8	2.750 ± 0.968
	val	4	3	2.000 ± 0.000
dysregulation	test	1	1	2.000 ± 0.000
	train	1	1	1.000 ± 0.000
	val	2	2	3.000 ± 1.000
epigenetic marker	test	2	1	2.000 ± 0.000
	train	0	0	0.000 ± 0.000
	val	0	0	0.000 ± 0.000
genetic susceptibility	test	3	3	3.000 ± 0.816
	train	23	17	3.261 ± 1.674
	val	0	0	0.000 ± 0.000
increased expression	test	42	21	1.786 ± 0.860
	train	141	67	2.078 ± 1.321
	val	30	18	1.967 ± 0.836
modulator decrease disease	test	2	2	2.000 ± 1.000
	train	3	3	2.333 ± 0.471
	val	0	0	0.000 ± 0.000
modulator increase disease	test	2	2	3.000 ± 0.000
	train	16	13	2.875 ± 1.111
	val	1	1	8.000 ± 0.000
negative prognostic marker	test	9	5	2.333 ± 0.471
	train	55	39	3.182 ± 1.280
	val	5	4	3.200 ± 0.980
no relation	test	1	1	3.000 ± 0.000
	train	15	14	5.467 ± 2.705
	val	1	1	2.000 ± 0.000
pathological role	test	10	10	3.100 ± 1.513
	train	46	38	2.804 ± 1.541
	val	9	8	2.556 ± 1.950
positive prognostic marker	test	4	2	3.000 ± 1.732
	train	3	3	3.333 ± 1.247
	val	0	0	0.000 ± 0.000
prognostic indicator	test	4	4	3.500 ± 1.658
	train	29	17	2.414 ± 0.617
	val	3	2	3.000 ± 1.414
relation undefined	test	19	13	1.684 ± 0.567
	train	35	19	1.714 ± 0.848
	val	9	8	2.444 ± 0.831
therapeutic target	test	4	4	2.000 ± 0.707
	train	36	17	2.222 ± 0.671
	val	7	7	3.571 ± 2.060
therapy resistance	test	5	3	1.200 ± 0.400
	train	21	13	2.857 ± 1.457
	val	5	4	2.000 ± 1.095

Supplementary data 7. Micro-average F1 scores for all tested models as the size of the fine-tuning training set increases. Reported F1 scores for fine-tuned models are averaged over five seeds. Dashed lines indicate the performance of zero-shot and few-shot prompted LLMs. LoRA fine-tuning of LLaMA 3.1 8B surpasses its zero- and few-shot variants once 20% of the fine-tuning data is used. With 60% of the training set, LLaMA 3.1 8B also exceeds the performance of the best 5-shot model (Claude 3.7).

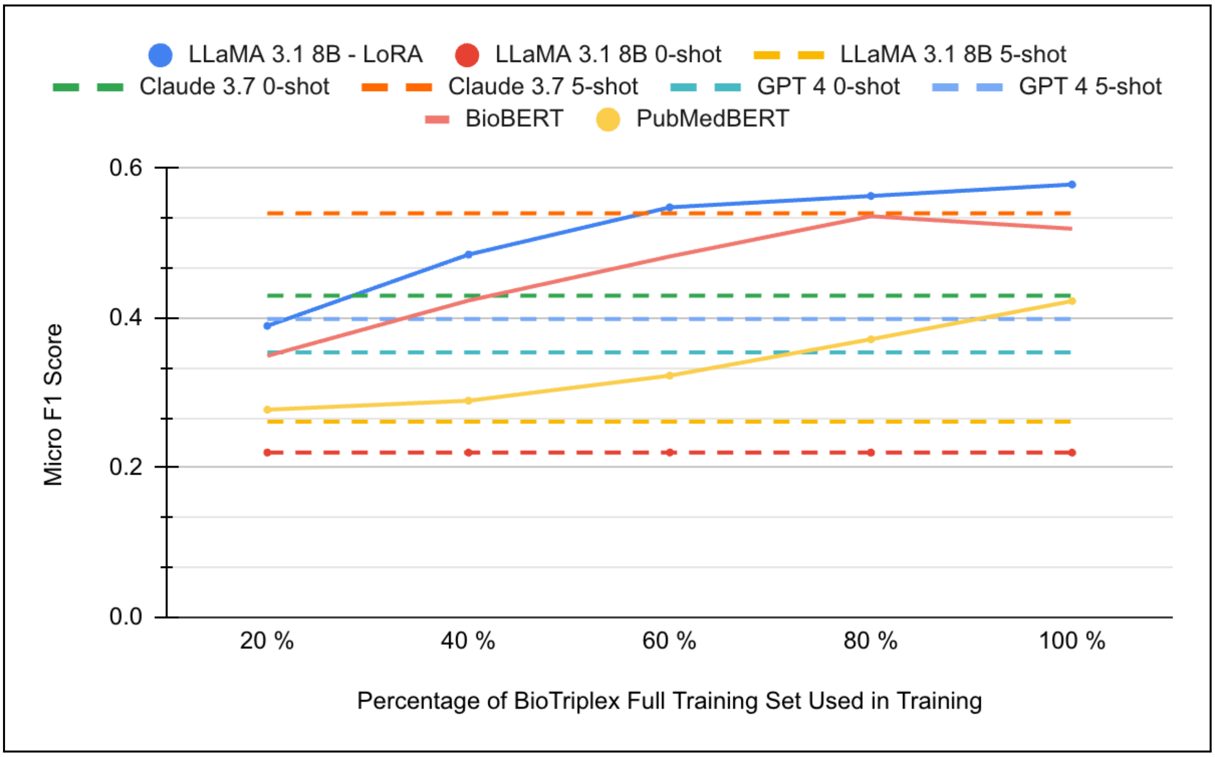

Supplement: btag037_Supplementary_Data [file btag037_supplementary_data.zip › BIOINF-2025-1732 Supplementary data REVISED.docx.pdf]
